# Genetic variation in the *hTAS2R38* taste receptor and food consumption among Finnish adults

**DOI:** 10.1007/s12263-014-0433-3

**Published:** 2014-10-11

**Authors:** Mari Sandell, Ulla Hoppu, Vera Mikkilä, Nina Mononen, Mika Kähönen, Satu Männistö, Tapani Rönnemaa, Jorma Viikari, Terho Lehtimäki, Olli T. Raitakari

**Affiliations:** 1 Functional Foods Forum, University of Turku, 20014 Turku, Finland; 2 Department of Biochemistry, University of Turku, Turku, Finland; 3 Department of Food and Environmental Sciences, University of Helsinki, Helsinki, Finland; 4 Research Centre of Applied and Preventive Cardiovascular Medicine, University of Turku, Turku, Finland; 5 Fimlab Laboratories, Department of Clinical Chemistry, School of Medicine, University of Tampere, Tampere, Finland; 6 Department of Clinical Physiology, Tampere University Hospital and University of Tampere, Tampere, Finland; 7 Department of Chronic Disease Prevention, National Institute for Health and Welfare, Helsinki, Finland; 8 Department of Medicine, University of Turku, Turku, Finland; 9 Division of Medicine, Turku University Hospital, Turku, Finland; 10 Department of Clinical Physiology and Nuclear Medicine, Turku University Hospital, Turku, Finland

**Keywords:** *TAS2R38*, Bitter taste, Vegetables, Adults

## Abstract

Genetic variation in bitter taste receptors, such as *hTAS2R38*, may affect food preferences and intake. The aim of the present study was to investigate the association between bitter taste receptor haplotypes and the consumption of vegetables, fruits, berries and sweet foods among an adult Finnish population. A cross-sectional design utilizing data from the Cardiovascular Risk in Young Finns cohort from 2007, which consisted of 1,903 men and women who were 30–45 years of age from five different regions in Finland, was employed. DNA was extracted from blood samples, and *hTAS2R38* polymorphisms were determined based on three SNPs (rs713598, rs1726866 and rs10246939). Food consumption was assessed with a validated food frequency questionnaire. The prevalence of the bitter taste-sensitive (PAV/PAV) haplotype was 11.3 % and that of the insensitive (AVI/AVI) haplotype was 39.5 % among this Finnish population. PAV homozygotic women consumed fewer vegetables than did the AVI homozygotic women, 269 g/day (SD 131) versus 301 g/day (SD 187), respectively, *p* = 0.03 (multivariate ANOVA). Furthermore, the intake of sweet foods was higher among the PAV homozygotes of both genders. Fruit and berry consumption did not differ significantly between the haplotypes in either gender. Individuals perceive foods differently, and this may influence their patterns of food consumption. This study showed that the *hTAS2R38* taste receptor gene variation was associated with vegetable and sweet food consumption among adults in a Finnish population.

## Introduction

The abundant consumption of a variety of vegetables, fruit and berries may provide protection from many chronic diseases (Boeing et al. 2012). However, the current levels of the consumption of vegetables and fruits in many European countries and the US are lower than recommended (Agudo et al. 2002; Guenther et al. 2006). In addition, the consumption of foods and beverages containing sugar may contribute to the development of obesity (Te Morenga et al. 2012). Taste is an important factor in food selection. Individual taste preferences may be partly genetically determined, which may explain differences in food consumption (Tepper 2008).

The abilities of individuals to recognize bitter compounds that contain a ‘–N–C=S’-structure, such as phenylthiocarbamide (PTC) and its chemical relative propylthiouracil (PROP), exhibit a bimodal distribution that distinguishes a sensitive and an insensitive phenotype (Kim and Drayna 2005). The degree of taste sensitivity for PTC and PROP has been linked to the TAS2R38 taste receptor haplotype (Kim et al. 2003). In general, three single-nucleotide polymorphisms (SNPs) within *hTAS2R38,* i.e., Ala49Pro, Val262Ala and Ile296Val, create numerous haplotypes. The Pro–Ala–Val (PAV) and Ala–Val–Ile (AVI) haplotypes are the most commonly found in European and Asian populations, while the Ala–Ala–Val (AAV) and Ala–Ala–Ile (AAI) haplotypes are uncommon (Kim and Drayna 2005; Kim et al. 2005). PAV homozygotes have been shown to be more sensitive to selected bitter-tasting compounds than are AVI homozygotes (Bufe et al. 2005). The perceived bitterness of different glucosinolate-producing vegetables, such as plants that belong to the *Brassicaceae* family, depends on *hTAS2R38* (Sandell and Breslin 2006). Moreover, the acceptance and consumption of vegetables and fruits have been linked to differences in sensitivity to PROP (Drewnowski et al. 1999; Dinehart et al. 2006; Kaminski et al. 2000; Yackinous and Guinard 2002). Few previous studies have reported on the connection between *hTAS2R38* genotype and vegetable consumption, and the results of these studies have inconsistently reported some small differences in total vegetable intake (Duffy et al. 2010), cruciferous vegetables intake (Sacerdote et al. 2007) or no difference in brassica vegetable intake (Gorovic et al. 2011). Furthermore, preferences for sweet foods may also be related to bitter taste sensitivity (Feeney 2011).

The *hTAS2R38* taste receptor genotype distribution varies between population groups (Mennella et al. 2010). Food culture and food consumption patterns also differ between countries. Gender differences in food consumption also prevail; Finnish women generally consume more vegetables and fruit than do men (Paturi et al. 2008). The objective of the present study was to investigate the distribution of *hTAS2R38* taste receptor haplotypes among adults in Finland [the Cardiovascular Risk in Young Finns Study (YFS) cohort]. Additionally, we primarily examined the associations of haplotypes, specifically the AVI and PAV homozygotes, with total consumptions of vegetables, fruits, berries and sweet foods. PAV/AVI group has been observed to be rather heterogeneous phenotypes linked to taste perception (Bufe et al. 2005). In this study, our special focus was on the extremes of bitter taste perception based on genetic variation without separate phenotyping in the sensory laboratory.

## Methods

### The Cardiovascular Risk in Young Finns Study

The YFS is a population-based follow-up study of the development of cardiovascular and other metabolic disorders in five study centers in Finland (Turku, Helsinki, Tampere, Kuopio and Oulu). The details of the study design and methods have been published previously (Raitakari et al. 2008). The first cross-sectional survey was conducted in 1980, and 3,596 children and adolescents aged 3, 6, 9, 12, 15 and 18 years were examined according to a standardized protocol. The present study is based on the data obtained at the follow-up study in 2007 when the subjects were 30–45 years of age. All subjects who participated in 1980 and still had a permanent address in Finland were invited, and 2,247 (63 %) participated. The subjects’ weights and heights were measured at the study visit, and the background characteristics were collected by questionnaire. The study was approved by the local ethics committees and was conducted following the guidelines of the Declaration of Helsinki. All participants provided written informed consent.

### Dietary assessment

Food consumption was assessed using a modified 131-item food frequency questionnaire (FFQ) developed and validated by the Finnish National Institute for Health and Welfare (Männistö et al. 1996; Paalanen et al. 2006). Using nine response categories that ranged from never to >6 portions/day, the participants were asked to report their habitual consumption of selected foods and dishes during the previous 12 months. The portion sizes were fixed in the questionnaire. These consumption figures were then used to calculate the average daily food consumptions using the latest version of the National Food Composition Database Fineli (National Public Health Institute 2007). The consumptions of selected foods and food groups were chosen for examination based on their nutritional roles in the diet and on previous knowledge of the effects the AVI/AVI and PAV/PAV haplotypes of the *TAS2R38* gene on food choices. Due to either generally low consumption and/or large individual variation in specific food consumption subgroups, only the total consumptions of each food group were statistically evaluated.

### Genotyping

The A49P (rs713598, G/G, C/G, C/C), A262 V (rs1726866, C/C, C/T, T/T) and V296I (rs10246939, G/G, G/A, A/A) alleles of the *hTAS2R38* gene were determined using allele-specific probes and primers from Applied Biosystems. Genomic DNA was extracted from EDTA-whole blood using QIAamp^®^ DNA Blood Midi Kits and the automated BioRobot M48 (Qiagen, Hilden, Germany). Genotyping was performed by using Taqman^®^ SNP Genotyping Assays (C_8876467_10 was used for the rs713598 assay, C_9506827_10 was used for the rs1726866 assay, and C_9506826_10 was used for the rs10246939 assay) and a ABI Prism 7900HT Sequence Detection System (Applied Biosystems, Foster City, CA, USA) according to manufacturers’ instructions. No discrepancies were detected in the genotyping results from duplicate analyses.

### Study population

The dietary information from the 2012 subjects was assessed. Questionnaires that were incompletely filled out or were unreliable (*n* = 16) were excluded, which led to the inclusion of 1,996 subjects. Genotyping was completed for 2,557 subjects. In total, the association analyses between the haplotypes and dietary intakes were carried out for 1,903 subjects, of which 1,055 (55 %) were women.

### Statistics

The food consumptions were energy adjusted using the residual method (Willett 2012), which is an established and a widely used method in nutritional epidemiology to enable the investigation of the quality, rather than the quantity, of foods in the diet. Energy-adjusted intake variables were computed as the residuals from the regression model, with total energy intake as the independent and the food consumption as the dependent variable. Multivariate analysis of variance (ANOVA) models were used to investigate the group differences in food consumption between the AVI/AVI and PAV/PAV genotypes, and an additive linear regression analysis was carried out to examine the allele-specific effects of the *hTAS2R38* genotypes. All models were adjusted for age, and the multivariate models were further adjusted for total energy and body mass index (BMI). The women and men were studied separately. All statistical analyses were performed with the SAS for Windows software package version 9.3 (SAS Institute Inc., Cary, NC).

## Results

The *hTAS2R38* haplotype distribution is shown in Table 1. No significant differences between the females and males were observed. The bitter taste-sensitive (PAV/PAV) haplotype accounted for 11.3 % of the sample, and the insensitive (AVI/AVI) haplotype accounted for 39.5 %. Across all subjects, 5.5 % carried at least one AAV haplotype.

**Table 1:** *hTAS2R38* haplotype distribution (%)

	Women (*n* = 1,378)	Men (*n* = 1,179)	All (*n* = 2,557)
PAV/AVI	42.3	45.2	43.6
AVI/AVI	40.5	38.4	39.5
PAV/PAV	11.7	10.8	11.3
AAV/AVI	3.0	3.3	3.1
PAV/AAV	2.5	2.2	2.3
AAV/AAV	0.1	0.1	0.1

There were no significant differences in any of the background variables between the AVI and PAV homozygote women and men (Table 2). Food consumptions, both in terms of total intake and specific subgroups (g/day) are presented in Table 3. The sweet food group included soft drinks, sweets, chocolate and ice cream.

**Table 2:** Background characteristics by gender and the PAV/PAV and AVI/AVI haplotypes of the *TAS2R38* gene

	Women		*p* value	Men		*p* value^a^
	AVI/AVI (*n* = 428)	PAV/PAV (*n* = 127)		AVI/AVI (*n* = 323)	PAV/PAV (*n* = 99)	
Age (years)	38.0 (30–45)	37.7 (30–45)	0.60	37.6 (30–45)	37.8 (30–45)	0.72
Marital status						
Unmarried	13 %	16 %		17 %	17 %	
Married or cohabiting	79 %	75 %		79 %	78 %	
Divorced or widowed	8 %	9 %	0.68	4 %	5 %	0.90
Education^b^						
Basic	36 %	36 %		49 %	47 %	
Intermediate	28 %	28 %		18 %	22 %	
High	36 %	36 %	0.98	33 %	31 %	0.91
Place of residence^c^						
Urban	60 %	53 %		61 %	58 %	
Rural	40 %	46 %	0.19	39 %	42 %	0.65
Body mass index (kg/m^2^)	25.5 (4.9)	25.4 (5.4)	0.98	26.6 (4.3)	27.0 (4.4)	0.41
Total energy intake (kJ/day)	9,142 (2,740)	9,105 (2,757)	0.80	11,355 (3,824)	11,232 (3,850)	0.78

The values are presented as the means and ranges (for age), standard deviations (for body mass index and total energy intake) or proportions (for marital status, education and place of residence)

^a^To examine the differences between the AVI/AVI and PAV/PAV groups, the *χ* ^2^ test was used for categorical variables, and the unpaired *t* test was used for continuous variables

^b^According to the highest level of education. Basic = elementary or vocational school; intermediate = intermediate school or institute; high = university or college

^c^Urban = town center or suburb; rural = countryside or village

**Table 3:** Consumption of selected foods (g/day) by gender and PAV/PAV, AVI/AVI and PAV/AVI haplotypes of the *TAS2R38* gene

	Women			Men		
	AVI/AVI (*n* = 428)	PAV/PAV (*n* = 127)	PAV/AVI (*n* = 440)	AVI/AVI (*n* = 323)	PAV/PAV (*n* = 99)	PAV/AVI (*n* = 375)
Vegetables	301 (187)	269 (131)	286 (176)	245 (186)	230 (133)	228 (155)
Leaf vegetables	17 (16)	13 (9)	15 (12)	12 (11)	12 (13)	12 (12)
Root vegetables	43 (35)	40 (25)	44 (34)	38 (35)	32 (19)	33 (29)
Cabbage	32 (35)	28 (27)	32 (33)	30 (46)	25 (26)	25 (25)
Onion	26 (17)	21 (11)	24 (16)	26 (18)	24 (12)	24 (12)
Fruit vegetables	169 (129)	150 (91)	160 (117)	122 (104)	119 (85)	123 (113)
Fruits and berries	261 (237)	269 (210)	259 (233)	170 (164)	180 (147)	157 (147)
Citrus fruits	66 (92)	74 (110)	63 (90)	30 (50)	38 (64)	32 (54)
Apples	83 (112)	81 (104)	79 (100)	40 (57)	46 (61)	41 (52)
Other fruits	82 (86)	82 (80)	82 (79)	75 (96)	69 (88)	60 (64)
Berries	27 (27)	28 (29)	30 (30)	22 (27)	22 (23)	17 (19)
Sweet foods	125 (168)	135 (142)	132 (128)	166 (232)	188 (244)	183 (224)
Soft drinks	87 (161)	89 (138)	90 (170)	130 (221)	145 (223)	144 (212)
Sweets	16 (16)	18 (19)	17 (17)	14 (17)	15 (20)	14 (15)
Chocolate	13 (15)	15 (16)	14 (15)	9 (12)	14 (17)	11 (13)
Ice cream	9 (12)	13 (16)	12 (16)	14 (29)	13 (14)	13 (19)

All values are presented as the means (standard deviations)

The multivariate ANOVA of the associations between haplotypes and food consumption (Table 4) revealed that the total intake of vegetables was lower among the PAV homozygotic women (*p* = 0.03). Both the PAV homozygotic women (*p* = 0.03) and men (*p* = 0.05) consumed more sweet foods than did the AVI homozygotic subjects. Fruit and berry consumption did not differ significantly between the genotypes (Table 4).

**Table 4:** Associations between food consumption and PAV/PAV and AVI/AVI haplotypes of the *TAS2R38* gene

	Women		Men		All	
	*β* (SE)	*p* value	*β* (SE)	*p* value	*β* (SE)	*p* value
Vegetables^a^	−0.08 (0.02)	0.03	−0.04 (0.03)	0.15	−0.06 (0.03)	0.10
Fruits and berries^b^	0.02 (0.08)	0.52	0.02 (0.07)	0.56	0.02 (0.05)	0.42
Sweet foods^c^	0.03 (0.01)	0.03	0.04 (0.01)	0.05	0.03 (0.01)	0.03

The values are presented as the standardized estimates and their standard errors for the differences in food consumption of the PAV/PAV genotype group compared with the AVI/AVI genotype group, which was used as the reference. Multivariate analysis of variance (ANOVA) adjusted for total dietary energy (kJ, residual method) and age (years)

^a^Leaf vegetables, root vegetables, cabbage, onion, fruit vegetables and other vegetables

^b^Citrus fruits, apples, other fresh fruits, canned fruits and berries

^c^Soft drinks, sweets, chocolate and ice cream

With the linear regression analyses, the results showed that the main finding in vegetable consumption (energy adjusted) in women remained significant (*p* = 0.04) when the analyzed data was adjusted for age, BMI (sex- and age-specific *z* score) and total energy (kJ/day). In other words, the difference between PAV/PAV women and AVI/AVI women was still significant in vegetable consumption. However, the difference in sweet foods intake was not significant anymore when data were adjusted for age, BMI and total energy at the same time.

The multivariate linear regression model was also used to analyze each of the three SNPs individually (Table 5). With energy-adjusted food consumption as the outcome and genotype (allele frequency) as a continuous predictor variable, the results showed that all the individual SNPs (Ala49Pro, Val262Ala and Ile296Val) were significant predictors of vegetable consumption in women. When additional controlling for total energy intake (kJ/day) and BMI (sex- and age-specific *z* score) were used, the effect remained significant in vegetable consumption in women with all the three SNPs. In the case of sweet food consumption, TAS2R28_A49P (rs713598) genotype was significant both in women (*p* = 0.03) and men (*p* = 0.04) (Model 1).

**Table 5:** Food consumption means (SD) by *TAS2R38* SNPs

Genotype	TAS2R38_49			TAS2R38_262			TAS2R38_296		
	GG	GC	CC	CC	CT	TT	GG	GA	AA
Women (*n*)	452	446	124	149	456	417	149	456	417
Men (*n)*	353	387	98	116	403	319	116	403	319
Vegetables (g/day)									
Women	298 (177)	279 (172)	265 (122)	267 (129)	281 (172)	296 (179)	267 (129)	281 (172)	296 (179)
*p* ^1^	0.01			0.04			0.04		
*P* ^2^	0.02			0.05			0.05		
Men	246 (172)	218 (155)	233 (124)	228 (120)	217 (151)	243 (175)	228 (120)	217 (151)	243 (175)
*p* ^1^	0.29			0.11			0.11		
*P* ^2^	0.54			0.25			0.25		
Fruits and berries (g/day)									
Women	259 (227)	259 (230)	270 (205)	269 (201)	255 (238)	262 (230)	269 (201)	255 (238)	262 (230)
*p* ^1^	0.61			0.71			0.71		
*P* ^2^	0.68			0.90			0.90		
Men	167 (157)	148 (152)	178 (144)	177 (141)	147 (151)	169 (162)	177 (141)	147 (151)	169 (162)
*p* ^1^	0.80			0.41			0.41		
*P* ^2^	0.82			0.48			0.48		
Sweet foods (g/day)									
Women	126 (158)	127 (188)	134 (140)	133 (135)	126 (184)	122 (162)	133 (135)	126 (184)	122 (164)
*p* ^1^	0.03			0.21			0.21		
*P* ^2^	0.15			0.28			0.28		
Men	163 (211)	182 (210)	189 (239)	189 (226)	182 (209)	166 (230)	189 (226)	182 (209)	166 (203)
*p* ^1^	0.04			0.12			0.12		
*P* ^2^	0.07			0.32			0.32		

^1^ *p* values from a multivariate linear regression analysis with energy-adjusted food consumption (residual method) as the outcome and genotype (number of risk alleles) as a continuous predictor variable, controlled for age (Model 1)

^2^Additional controlling for total energy intake (kJ/day) and BMI (sex- and age-specific *z* score) (Model 2)

## Discussion

This is the first study to report the *hTAS2R38* haplotype distribution of a large-sample adult Finnish population. Worldwide variation in this distribution seems to depend on race/ethnicity. In a study by Mennella et al. (2010), the prevalence of PAV/PAV in the Caucasian population was found to be 18 %; in the African-American population, the prevalence was 16 %; and in the other population (i.e., Asian, Hispanic and mixed ancestry), the prevalence was 22 %. In Europe, the proportion of PAV/PAV in a German sample was found to be 14.5 % (Sausenthaler et al. 2009), and in a genetically isolated population in Southern Italy, this proportion was found to be 22.5 % (Tepper et al. 2008). Thus, the proportion of PAV homozygotes seems to be lower in Finland (11 %). The number of people carrying at least one AAV haplotype was relatively high in the Finnish population compared with the general statistics related to *hTAS2R38* haplotype distributions (Kim and Drayna 2005).

The results of the present study are in accordance with previous findings that have shown that persons with the bitter taste-sensitive *hTAS2R38* haplotype consume fewer vegetables. In Italy, Sacerdote et al. (2007) reported small differences in cruciferous vegetable intake between different *hTAS2R38* genetic variants, and one study with a small sample found differences in total vegetable intake among college-aged adults in the US (Duffy et al. 2010). The flavor characteristics of many vegetables, particularly brassica vegetables that contain bitter-tasting compounds (Zabaras et al. 2013), may differ substantially. In the present study, based on the linear regression analyses, separately carried out for the three examined SNPs of the *hTAS2R38* gene, we found that the vegetable consumption differences observed between PAV/PAV and AVI/AVI women can be explained with all of the three polymorphisms. This shows that all the three SNPs must be determined when investigating the association between *hTAS2R38* and vegetable consumption.

Vegetable sweetness has been shown to be a significant predictor of preference for vegetables, and those who taste PROP as the most bitter also perceive these vegetables to be the most bitter and least sweet (Dinehart et al. 2006). However, studies of PROP tasting have not always produced consistent results. Differences in the actual procedures concerning the classifications of tasters, supertasters and nontasters of PROP may partially explain the contradictory results. The use of haplotypes rather than taste perception-related phenotypes may provide more precise specification. We wanted to reach a larger study population with genotyping and, therefore, did not perform PROP testing in the sensory laboratory in the present study.

No significant differences in the consumptions of fruits and berries between genotypes were observed in the present study. Previous studies have reported differences in the taste preferences of bitter taste-sensitive and insensitive subjects, for example, differences have been reported regarding preferences for grapefruit (Drewnowski et al. 1997) and some berries (Laaksonen et al. 2013). Most fruits and berries can be characterized mainly by sweet or sour tastes, but bitterness and astringency (Laaksonen et al. 2010, 2013) may also be important sensory properties of the sensory profiles of these foods that potentially limit consumption (Sandell et al. 2012).

In the present study, the total consumption of specific sweet foods was higher among the PAV homozygotes in both genders when energy-adjusted data were controlled for age. However, with further adjustments for BMI and total energy, difference in sweet consumption became weaker. Based on the Table 2, unstandardized BMI did not differ between haplotypes. This finding may suggest that complex interactions between BMI, total energy intake and food consumption are difficult to interpret in multivariate models. The correlation between BMI and sweet food consumption or total energy and sweet food consumption were higher among PAV homozygotes than AVI homozygotes (data not shown). In addition, BMI may have a role in the possible causal chain with taste preferences, food consumption and energy intake and be in association with possible food reporting bias (Ferrari et al. 2002). Therefore, BMI-adjusted results should be interpreted with caution.

Previous studies have shown that, among adults, preferences for sweetness in sensory laboratory settings may differ, and bitter taste-sensitive subjects (as defined by PROP tasting) have been reported more likely to be sweet dislikers (Yeomans et al. 2007). Shafaie et al. (2013) found among young, lean females that PROP supertasters consumed less sweet foods (sweet food group including cakes, cookies and pastries) than nontasters or medium tasters when food intake was measured in laboratory setting. Moreover, gender differences have been reported, for example, the liking of sweet foods decreases with PROP bitterness among women but tends to increase among men (Duffy and Bartoshuk 2000). PAV children have been found to prefer cereals and beverages with higher sugar contents, while no such association between haplotype and sweet preference has been observed in adult women (Mennella et al. 2005). We found among preschool-aged Finnish children that PAV homozygote boys consumed more sugar and candy based on their actual food consumption data of 7-day food records (Hoppu et al. 2014). Thus, there are some discrepancies between studies regarding the preferences or consumption of sweet foods between PROP taster groups and TAS2R38 genotypes, which could be explained by the different subject characteristics, data collection and analysis methods, and contents of food groups. To our knowledge, differences in the habitual consumption of sweet foods between different *hTAS2R38* haplotypes have not previously been reported among adults.

In addition to the differences in genetic backgrounds, the vegetable consumption patterns of different countries may also depend on, for example, the availability and affordability of vegetables and food culture (Prättälä et al. 2009). Individuals choose their foods based on numerous factors that are both conscious and unconscious. The effects of taste preferences may be overpowered by strong health-related, cultural, psychological or economic factors in some circumstances. When comparing results across distinct populations, it must be acknowledged that the relative contributions of different factors to individuals’ food choices may differ with age, gender and sociodemographic status.

The FFQ used in this study was used in a nationally representative study and has been validated against food records (Paalanen et al. 2006). However, small differences in the actual consumption of specific vegetables, fruit or berries may remain undetected with the FFQ method. The FFQ is used to estimate long-term food consumption, and its accuracy is compromised by the use of a predefined food list. Therefore, the food groupings may have diluted possible differences in the consumption of particular food items. In addition to quantity, future studies should evaluate the variety of fruit and vegetable intake (Cooper et al. 2012).

In conclusion, we found that the consumption of vegetables was lower and that of sweet foods was higher among bitter taste-sensitive Finnish adults. Both of these behaviors may be related to generally more unhealthy dietary patterns and thus also be related to cardiovascular risk factors (Mikkilä et al. 2007). Whether these small differences in vegetable and sweet food consumption between haplotypes have long-term health effects remain to be further studied.
